# Targets and Candidate Agents for Type 2 Diabetes Treatment with Computational Bioinformatics Approach 

**DOI:** 10.1155/2014/763936

**Published:** 2014-10-21

**Authors:** Qiong Wang, Zhigang Zhao, Jing Shang, Wei Xia

**Affiliations:** Department of Endocrinology, Henan Provincial People's Hospital, No. 7 Weiwu Road, Zhengzhou 450003, China

## Abstract

We sought to explore the molecular mechanism of type 2 diabetes (T2D) and identify potential drug targets and candidate agents for T2D treatment. The differentially expressed genes (DEGs) were assessed between human pancreatic islets with T2D and normal islets. The dysfunctional pathways, the potential transcription factor, and microRNA targets were analyzed by bioinformatics methods. Moreover, a group of bioactive small molecules were identified based on the connectivity map database. The pathways of Eicosanoid Synthesis, TGF-beta signaling pathway, Prostaglandin Synthesis and Regulation, and Integrated Pancreatic Cancer Pathway were found to be significantly dysregulated in the progression of T2D. The genes of *ZADH2* (zinc binding alcohol dehydrogenase domain containing 2), *BTBD3* (BTB (POZ) domain containing 3), Cul3-based ligases,  *LTBP1* (latent-transforming growth factor beta binding protein 1), *PDGFRA* (alpha-type platelet-derived growth factor receptor), and *FST* (follistatin) were determined to be significant nodes regulated by potential transcription factors and microRNAs. Besides, two small molecules (sanguinarine and DL-thiorphan) were identified to be capable of reverse T2D. In the present study, a systematic understanding for the mechanism underlying T2D development was provided with biological informatics methods. The significant nodes and bioactive small molecules may be drug targets and candidate agents for T2D treatment.

## 1. Introduction

Type 2 diabetes (T2D) is a chronic metabolic disorder, which results from impaired insulin secretion and action in target tissues [1, 2]. Currently, the incidence of T2D is increasing worldwide [3]. And it is reported that there will be 280 million cases suffering from T2D in 2011 [4]. The prevalence trend is considered to be ascribed to genetic variants and environmental factors such as sedentary lifestyle, obesity [3, 5–7]. Despite the foundational evidence of the mechanism underlying T2D is far from being clear, great contributions have been made to address this health concern.

The variants of some critical genes are determined to contribute to T2D development. The TCF7L2 gene of transcription factor 7-like 2 commonly variant in individuals confers the risk of suffering from T2D [8]. Other genes that have expression variation in patients with T2D are indicated to be* CAPN10 *(calpain 10),* KIR6. 2* (potassium inward-rectifier 6.2),* PPAR γ* (peroxisome proliferator-activated receptor *γ*), and* IRS-1 *(insulin receptor substrate-1) [9]. Another important understanding of the mechanism underlying T2D is associated with the dysfunction of *β*-cell in human pancreatic islets [10, 11]. The decreased *β*-cell mass and increased *β*-cell apoptosis resulted in T2D development and progression. The discovery of novel approaches for T2D treatment has concerned the uncharted area underlying mechanism.

In this work, we downloaded the microarray gene expression data of human pancreatic islets with or without T2D from GEO database. A comprehensive perspective was provided to understand the mechanism underlying T2D with the application of computational bioinformatics method. The dysfunction pathways, potential transcription factor targets, and microRNA targets were explored based on DEGs analysis. Besides, the candidate small molecules were identified, which were capable of ameliorating these genetic changes.

## 2. Data and Methods

### 2.1. Affymetrix Microarray Data and Differentially Expressed Genes Analysis

The cDNA microarray expression data (GSE38642) was downloaded from Gene Expression Omnibus (GEO) database (http://www.ncbi.nlm.nih.gov/geo/), which was deposited by Taneera et al. [4]. The gene expression data were collected from human pancreatic islets including 54 nondiabetic samples and 9 T2D samples. As the progression of T2D is strongly associated with HbA1c expression [4], we only selected the 29 samples without T2D (HbA1c expression < 6.0) in control group and 8 samples with T2D (HbA1c expression > 6.0) in experimental group. We downloaded the raw data and annotation files for further analysis based on the platform of GPL6244 (Affymetrix Human Gene 1.0 ST Array).

Geoquery software is a tool for analysis and comprehension of microarray and genomics data directly from GEO database [12]. Limma statistics is commonly used for assessing differential expression genes [13, 14].

The microarray data was further performed by Geoquery in *R* statistical programming environment [15]. Then the differentially expressed genes between type 2 diabetic islets and nondiabetic islets were analyzed by limma package and were tested by modified *t*-test based on Empirical Bayes Methods [16].

### 2.2. Pathways Enrichment Analysis of Differentially Expressed Genes

WikiPathways is a public wiki for building research communities on biological pathways, which is characterized for pathway curation and pathway ontology annotations [17]. WebGestalt2 is a gene set analysis toolkit for functional enrichment analysis for large scale of genome [18].

We collected all the metabolic and nonmetabolic pathways from WikiPathways database and performed pathway enrichment analysis with the application of Gene Set Analysis Toolkit V2.

### 2.3. Prediction of Potential Transcription Factors Targets and MicroRNAs for Differential Expression Genes

Molecular Signatures Database (MSigDB) is freely available (http://www.broadinstitute.org/gsea/msigdb/index.jsp) collection of a large scale of well-annotated genomic data [19].

The entire set of transcription factor target gene signatures and microRNA data were obtained from the MSigDB. The gene set enrichment analysis was performed on hypergeometric algorithm. Finally, the potential transcription factors targets and microRNAs were obtained after testing by BH (Barnes-Hut) algorithm.

### 2.4. The Construction of Regulatory Network

We integrated the data of DEGs, potential transcription factor binding sites, and microRNAs obtained in our work and established the regulatory network. And we also constructed a regulatory motif with the DEGs regulated by multiple transcription factors and microRNAs for further analysis.

### 2.5. Identification of Candidate Small Molecules

The connectivity map (CMap) deposited genome-wide transcriptional expression data (7056 gene expression profiles) from 6100 small molecules treatment-control experiments [20].

We firstly divided the DEGs identified in our paper into two groups: upregulated DEGs and downregulated ones and selected the significantly differential expression genes (Top 500) in each group. The gene set enrichment analysis (GSEA) was performed between the significantly differential expressed genes and those from treatment-control pairs in CMap database. Then an enrichment score ranging from −1 to 1 was obtained, which represented the level of similarity. When the positive enrichment score was closed to 1, the corresponding bioactive small molecule (perturbagen) was considered to reversal the expression of query signature in the progression of disease, otherwise the perturbagen contributed to the development of disease.

## 3. Results

### 3.1. Identification of Differentially Expressed Genes

To assess the differentially expressed genes, we downloaded the GSE38642 gene expression profile from GEO database. After analyzed by limma package and *t*-test, we defined *P* < 0.0001 as the cutoff value. Total 225 genes were identified to be significantly differential expressed between T2D islets tissues and normal tissues.

### 3.2. Identification of Dysfunction Pathways

In order to investigate the DEGs in molecular functional level, we carried out pathway enrichment analysis based on WikiPathways database. Total of 15 pathways were revealed to be significantly dysregulated with *P* < 0.05 and at least 2 genes enriched.

As shown in Table 1, the enriched pathways terms relevant with cell surface function, signal transduction, hormone regulation, cellular metabolism, and immune response were determined to be dysregulated in the progression of T2D, such as focal adhesion, MAPK signaling pathway, Prostaglandin Synthesis and Regulation, Eicosanoid Synthesis, Mitochondrial LC-Fatty Acid, Beta-Oxidation, Selenium Pathway, Fatty Acid Biosynthesis, Tryptophan metabolism, IL-6 signaling pathway, IL-7 signaling pathway, IL-1 signaling pathway, Inflammatory Response Pathway, and Complement and Coagulation Cascades. Besides, the Integrated Pancreatic Cancer Pathway was also identified to be disturbed in T2D development.

### 3.3. The Potential Transcription Factor Targets and MicroRNAs

The changes in the patterns of gene expression were affected by transcriptional regulation and posttranscriptional regulation; so we predicted the potential transcription factor targets and microRNA targets to further explore the mechanism underlying T2D progression.

After investigation by hypergeometric and BH algorithm, we defined *P* < 10^−10^ and *P* < 10^−6^ as threshold values in transcription factor targets analysis and microRNAs targets analysis, respectively.

As shown in Table 2, the enrichment transcription factor targets were explored based on the upstream sequences of DEGs. And the significant microRNAs and targets uncovered in this work were listed in Table 3.

### 3.4. The Regulatory Network Construction

**Figure 1:**
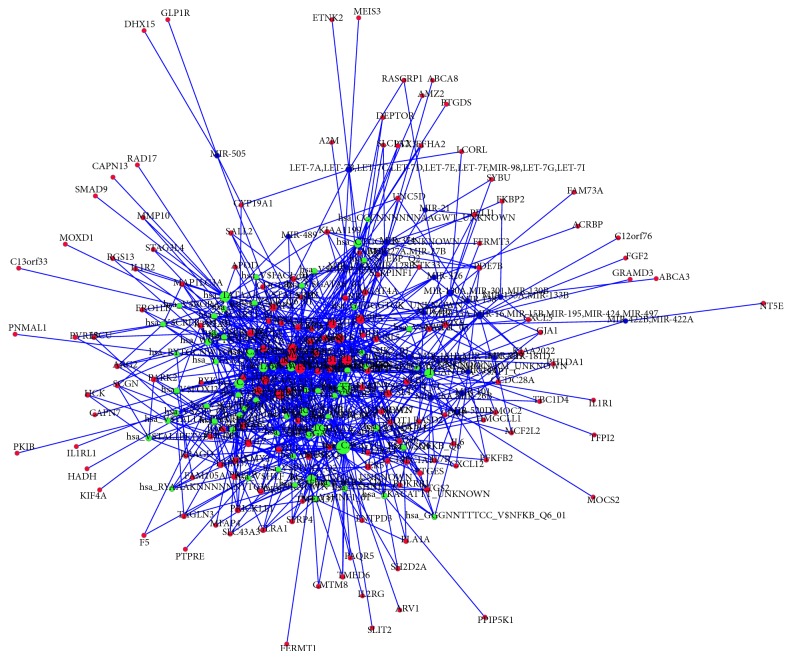
The regulatory network of DEGs by transcription factors and microRNAs. Red notes: DEGs; green nodes: transcription factor targets; blue nodes: microRNA targets; edges: interactions between two nodes. The bigger nodes indicate to have more interactions with others.

**Figure 2:**
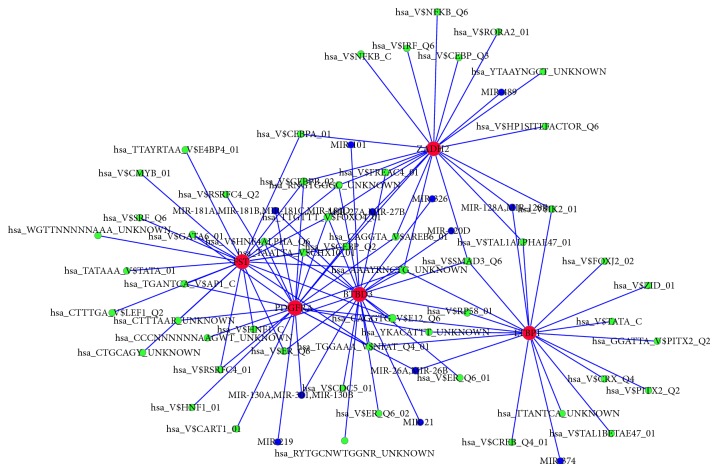
Regulatory motif of DEGs by transcription factors and microRNAs. Red notes: DEGs; green nodes: transcription factor targets; blue nodes: microRNA targets. The bigger nodes indicate to have more interactions with others.

To investigate the associations between DEGs and microRNAs, transcription factors, we constructed the regulatory network. As shown in Figure 1, different DEGs were regulated by different microRNAs and transcription factors. The DEGs involved with multiple regulators might play key roles in the progression of T2D; therefore we selected the DEGs corresponding to multiple microRNAs and transcription factors (*n* ≥ 20) to establish the regulatory motif.  Figure 2 showed that 5 genes played critical roles in the T2D development, including* ZADH2, BTBD3, LTBP1*,* PDGFRA, *and* FST*.

### 3.5. Identification of Candidate Small Molecules

We performed computational bioinformatics analysis to identify the candidate drugs for T2D treatment. After comparing the query signatures induced by DEGs with data from CMap database, a large amount of small molecules was identified, which had positive or negative correlation to query signature. The top 20 small molecules closely relevant with T2D were listed in Table 4. The small molecules with higher positive enrichment scores were determined to be sanguinarine (enrichment score = 0.977) and DL-thiorphan (enrichment score = 0.956). In addition, small molecule of felbinac showed highly significant negative score (enrichment = −0.847).

## 4. Discussion

Nowadays, T2D is highlighted by its increasing epidemicity all over the word [3]. Although numerous studies have been conducted concerning the therapies for T2D, the effective approaches for T2D treatment are relatively rare. The current work provided the foundational evidences for T2D development with systematic informatics analysis. In this paper, we downloaded the microarray gene expression data (GSE38642) from GEO database and identified the DEGs between diabetic and nondiabetic human islets. Results showed that, using the cutoff value of *P* < 0.0001, total 225 genes were differentially expressed. By pathway enrichment analysis of the DEGs, 15 pathways were revealed to be significantly dysregulated such as Eicosanoid Synthesis, Prostaglandin Synthesis and Regulation, and Integrated Pancreatic Cancer Pathway.

Eicosanoid is a critical signaling molecules biological process and played diverse and complex roles in biological and pathological control [21]. Eicosanoids consist of multiple subfamilies including prostaglandins, thromboxanes, leukotrienes, and derivatives of arachidonate [22]. Many diseases such as cardiovascular disease [23], inflammatory bowel disease [24], and diarrhoeal diseases [25] were mediated by the secretion of eicosanoids. As outlined in previous study, eicosanoids played key roles in modulating platelet function of T2D patients. Thromboxane, served as a member of eicosanoid family, can induce platelet aggregation to vascular endothelium resulting in platelet dysfunction [26]. Platelet aggregation suppressed the normal interaction of intact healthy vascular endothelium with platelets, which might result in macrovascular and microvascular events T2D patients.

Prostaglandin is also a member of eicosanoids, deriving from unsaturated fatty acids [27]. The renal production of prostaglandins has been reported to be associated with nephropathy in T2D [28]. The expression of prostaglandins and their corresponding receptors induced in islets is revealed to be contributors of T2D development [29]. The expression of prostaglandin E2 (PGE2) was elevated, which was positively related with the activation of prostaglandin E receptor 3 (EP3). The activation of PGE2-to-EP3 signaling pathway resulted in the decline of the cAMP activation and insulin secretion induced by glucose. The accumulation of EP3 and PGE2 production contributed to T2D development and *β*-cell dysfunction. Thus, the pathways related with Eicosanoid Synthesis and Prostaglandin Synthesis and Regulation played crucial roles in T2D development and progression. Besides, Integrated Pancreatic Cancer Pathway was also indicated to be a significant pathway in T2D development. Although there were few evidences concerning the association between T2D and integrated pancreatic cancer, it implied that T2D might be a precipitating factor for patients suffering from integrated pancreatic cancer.

Our results also showed that the genes of* LTBP1*,* PDGFRA*, and* FST *were the most significant targets for potential transcription factors and microRNAs. Among these significant targets,* LTBP1 *encoded for latent-transforming growth factor beta binding protein 1 which is a member of carrier proteins [30]. LTBP1 has various interactions with extracellular matrix proteins and TGF-beta (TGF-*β*) [31]. TGF-*β* signaling pathway showed tightly association with diabetes development. It is reported that the level of glucose has a direct effect on TGF-*β* activation [32]. An elevated expression of TGF-*β* was observed in serum of patients with T2D and antidiabetic treatment was able to reverse this trend [33]. Another report suggested that the suppression of TGF-*β*-TGF-*β* receptor interaction is available for preventing diabetes progression by inhibiting the differentiation of islet-reactive CD8^+^ T cells in type 1 diabetes [34]. By pathway enrichment analysis, our results also showed that TGF-*β* signaling pathway was significant in the T2D progression.

In addition,* PDGFRA* encoded alpha-type platelet-derived growth factor receptor is one of the latent TGF-beta binding proteins [35]. The production of* PDGFR* is considered to be interacted with PI3K p85*α* and PI3Kp85*α*pY580 is activated by insulin receptor tyrosine kinase [36–38].* FST* is the gene for follistatin which also served as activin-binding protein. Follistatin generally exists in blood and is considered to be involved in the inflammatory response stimulated by tissue injury or pathogenic incursion. Despite the clarification of mechanism underlying T2D concerning* PDGFRA* and* FST* was far from being clear, the significant nodes in regulatory networks may be potential drug targets for T2D treatment.

Besides, another important implication in our work was the identification of a group of small molecules. Data in Table 4 showed that the small molecules of sanguinarine (enrichment = 0.977) and DL-thiorphan (enrichment = 0.956) showed highly significant positive scores, suggesting that these small molecules are candidate agents targeting for T2D.

Sanguinarine is a benzophenanthridine alkaloid, which has been ascribed to a novel bioactive component extracted from plants [39]. And it has showed various properties including antimicrobial, antioxidant, and anti-inflammatory [40]. Previous researches proved that sanguinarine possessed potent anticancer activity against many different tumors, such as gastric osteosarcoma adenocarcinoma [41], osteosarcoma [42], prostate tumor [43], and oral cancers [44]. Sanguinarine prevented the development of cancers by inducing cancer cell apoptosis, suppressing tumor growth, migration, and invasion [45, 46]. A present study revealed that sanguinarine is involved in cell migration and angiogenesis suppression in cancer development by inhibiting the activity of vascular endothelial growth factor (VEGF) [39]. In spite of the increasing studies highlighting the anticancer property of sanguinarine, reviews also indicated the sanguinarine antidiabetic activity [47]. Sanguinarine derived from* Fumaria parviflora* plants has a hypoglycemic effect. In addition, sanguinarine has been used as an important drug against infections in one or more countries worldwide [48]. Moreover, DL-thiorphan is served as the specific neutral endopeptidase (NEP) inhibitor, which is widely used to differentiate NEP enzyme activity. NEP enzyme is a membrane-bound metallopeptidase that plays key roles in wound repair [49]. Fatty acids and glucose stimulated the expression of NEP. The activity of NEP was increased in the skin of objects with diabetic wound [50]. However, there are insufficient evidences indicating DL-thiorphan can be directly used in glucose control for patients with T2D. Therefore, sanguinarine and DL-thiorphan may be candidate agents for diabetes treatment in the near future.

In summary, the present study provides a systematic understanding for the mechanism underlying T2D development. The significant nodes such as* LTBP1*,* PDGFRA*, and* FST* assessed in regulatory network may be drug targets for T2D treatment. And sanguinarine and DL-thiorphan may be candidate agents targeting for T2D. However, more studies are required to confirm these discoveries in our work.

## Figures and Tables

**Table 1:** Dysfunction pathways between human T2D islet cells and normal islet cells.

Pathway	Count	*P* value
Eicosanoid synthesis	3	0.0016
MAPK signaling pathway	7	0.0029
IL-6 signaling pathway	4	0.005
Integrated Pancreatic Cancer Pathway	7	0.0055
Mitochondrial LC-Fatty Acid Beta-Oxidation	2	0.0152
Complement and Coagulation Cascades	3	0.0205
Focal adhesion	6	0.021
Selenium Pathway	4	0.0316
IL-7 signaling pathway	2	0.0354
TGF-beta signaling pathway	3	0.0362
IL-1 signaling pathway	3	0.0377
Fatty Acid Biosynthesis	2	0.0436
Tryptophan metabolism	3	0.0441
Inflammatory Response Pathway	2	0.0464
Prostaglandin Synthesis and Regulation	2	0.0494

**Table 2:** Potential transcription factor targets.

TF target	*P* value	TF target	*P* value
hsa_V$RP58_01	9.61*E* − 05	hsa_V$SRF_Q6	0.0192
hsa_GGARNTKYCCA_UNKNOWN	0.0002	hsa_V$PITX2_Q2	0.0201
hsa_TTGTTT_V$FOXO4_01	0.0008	hsa_V$IRF_Q6	0.0201
hsa_V$CART1_01	0.0009	hsa_V$HNF1_C	0.0205
hsa_RYTGCNWTGGNR_UNKNOWN	0.0019	hsa_V$CEBPA_01	0.0205
hsa_TTAYRTAA_V$E4BP4_01	0.0021	hsa_GGGNNTTTCC_V$NFKB_Q6_01	0.0209
hsa_V$NFKB_Q6	0.0024	hsa_V$TAL1BETAE47_01	0.0209
hsa_RYAAAKNNNNNNTTGW_UNKNOWN	0.0027	hsa_V$CMYB_01	0.0214
hsa_V$NFKB_C	0.0027	hsa_V$HLF_01	0.0214
hsa_TATAAA_V$TATA_01	0.0041	hsa_V$CDC5_01	0.0218
hsa_V$CEBP_Q2	0.0052	hsa_V$TAL1ALPHAE47_01	0.0223
hsa_CTTTAAR_UNKNOWN	0.0054	hsa_V$RSRFC4_01	0.0223
hsa_V$FOXJ2_02	0.0057	hsa_V$CEBP_Q3	0.0232
hsa_V$SMAD3_Q6	0.0058	hsa_V$ICSBP_Q6	0.0252
hsa_TGGAAA_V$NFAT_Q4_01	0.0063	hsa_V$ZID_01	0.0267
hsa_CAGGTA_V$AREB6_01	0.0064	hsa_CCCNNNNNNAAGWT_UNKNOWN	0.0269
hsa_YTAAYNGCT_UNKNOWN	0.0071	hsa_V$RORA2_01	0.0291
hsa_V$FREAC4_01	0.0079	hsa_CAGGTG_V$E12_Q6	0.0293
hsa_V$ER_Q6_02	0.0084	hsa_V$GATA6_01	0.031
hsa_V$CEBPB_02	0.0092	hsa_V$E4BP4_01	0.0315
hsa_AAAYRNCTG_UNKNOWN	0.0096	hsa_V$CREB_Q4_01	0.0321
hsa_TAATTA_V$CHX10_01	0.0101	hsa_V$IK2_01	0.0328
hsa_TTANTCA_UNKNOWN	0.0106	hsa_V$CRX_Q4	0.0334
hsa_V$HNF4ALPHA_Q6	0.0113	hsa_GGATTA_V$PITX2_Q2	0.0347
hsa_V$ER_Q6_01	0.0118	hsa_V$ER_Q6	0.0352
hsa_CTGCAGY_UNKNOWN	0.0129	hsa_V$RSRFC4_Q2	0.0364
hsa_RNGTGGGC_UNKNOWN	0.0133	hsa_V$TATA_C	0.0371
hsa_V$HP1SITEFACTOR_Q6	0.0134	hsa_V$FAC1_01	0.0378
hsa_TGANTCA_V$AP1_C	0.0141	hsa_YKACATTT_UNKNOWN	0.0384
hsa_TGTYNNNNNRGCARM_UNKNOWN	0.0153	hsa_V$GATA1_05	0.0412
hsa_V$HNF1_01	0.018	hsa_WGTTNNNNNAAA_UNKNOWN	0.0413
hsa_CTTTGA_V$LEF1_Q2	0.0183	hsa_GTGGGTGK_UNKNOWN	0.0485

**Table 3:** Potential microRNA targets.

Target sequence	MicroRNAs	*P* value
hsa_TATTATA	MIR-374	0.0018
hsa_TGAATGT	MIR-181A, MIR-181B, MIR-181C, MIR-181D	0.0068
hsa_TTGCACT	MIR-130A, MIR-301, MIR-130B	0.0069
hsa_GGGACCA	MIR-133A, MIR-133B	0.0139
hsa_ATGTCAC	MIR-489	0.016
hsa_TGCTGCT	MIR-15A, MIR-16, MIR-15B, MIR-195, MIR-424, MIR-497	0.0174
hsa_GTTTGTT	MIR-495	0.0209
hsa_TACTTGA	MIR-26A, MIR-26B	0.0245
hsa_GACAATC	MIR-219	0.0262
hsa_GTGTTGA	MIR-505	0.0269
hsa_TCATCTC	MIR-143	0.0269
hsa_ATACTGT	MIR-144	0.0283
hsa_GTACTGT	MIR-101	0.0283
hsa_CCCAGAG	MIR-326	0.0322
hsa_CTACCTC	LET-7A, LET-7B, LET-7C, LET-7D, LET-7E, LET-7F, MIR-98, LET-7G, LET-7I	0.0346
hsa_ATAAGCT	MIR-21	0.0391
hsa_CACCAGC	MIR-138	0.0393
hsa_TTTGTAG	MIR-520D	0.0394
hsa_CACTGTG	MIR-128A, MIR-128B	0.0419
hsa_ACTGTGA	MIR-27A, MIR-27B	0.0426
hsa_AAGTCCA	MIR-422B, MIR-422A	0.0458

**Table 4:** Top 20 significant small molecules.

CMap name	Enrichment	*P*
8-Azaguanine	0.932	0.00004
Apigenin	0.86	0.00052
Chrysin	0.931	0.00056
Sulfametoxydiazine	0.855	0.00056
Lycorine	0.803	0.00068
Digoxin	0.846	0.0008
Prochlorperazine	0.467	0.00086
Sanguinarine	0.977	0.00087
Helveticoside	0.733	0.00097
Felbinac	−0.847	0.00101
Adiphenine	−0.771	0.00118
Diloxanide	−0.83	0.00157
Etiocholanolone	−0.704	0.00157
Heptaminol	−0.753	0.0017
Acetylsalicylic acid	0.499	0.00174
Proscillaridin	0.903	0.00182
Cinchonine	−0.82	0.00197
0316684-0000	−0.813	0.00229
Proadifen	0.806	0.00265
DL-Thiorphan	0.956	0.00342
